# Fatal Disseminated Strongyloidiasis in a Patient With Untreated HIV: A Diagnostic Challenge

**DOI:** 10.14309/crj.0000000000002246

**Published:** 2026-07-20

**Authors:** Gedion Yilma Amdetsion, Robert Kwei-Nsoro, Cherechi Nwabeuze, Ayokunle Abegunde

**Affiliations:** 1 Department of Medicine, Cook County Hospital, Chicago, IL; 2 Division of Gastroenterology and Hepatology, Cook County Hospital, Chicago, IL

**Keywords:** strongyloides stercoralis, disseminated strongyloidiasis, hyperinfection syndrome, HIV, eosinophilia

## Abstract

*Strongyloides stercoralis* hyperinfection carries near-universal mortality if untreated. We present a 52-year-old man from Central America with advanced HIV (CD4 196 cells/μL) who presented with chronic diarrhea, vomiting, and weight loss. Cross-sectional imaging revealed small bowel thickening. Endoscopy demonstrated gastric and duodenal mucosal friability and ulceration. His course was complicated by polymicrobial Gram-negative bacteremia and meningitis. Retrospective identification of antecedent eosinophilia prompted suspicion for *Strongyloides*, confirmed on intestinal biopsies. The patient died before treatment could be initiated. This case underscores the importance of early clinical suspicion and empiric treatment in high-risk patients.

## INTRODUCTION

*Strongyloides stercoralis* is a soil-transmitted nematode infecting over 100 million people worldwide, predominantly in tropical regions.^1^ Its unique autoinfective life cycle allows persistence within the human host for decades.^1,2^ Although chronic infection is typically subclinical, immunocompromised hosts, particularly those receiving corticosteroids or coinfected with human T-lymphotropic virus type 1 (HTLV-1), can develop hyperinfection syndrome, wherein migrating larvae carry enteric organisms into the bloodstream, producing polymicrobial bacteremia and meningitis.^3–5^ We present a case of fatal disseminated strongyloidiasis in a patient with newly diagnosed, untreated HIV, highlighting the diagnostic challenges and critical importance of early clinical suspicion.

## CASE REPORT

A 52-year-old man originally from Central America with newly diagnosed HIV infection and type 2 diabetes mellitus presented with a 2-week history of nausea, cyclical vomiting (4 episodes/d), watery diarrhea (5–6 episodes/d), low-grade fever, diffuse abdominal pain, and an unintentional 20-pound weight loss. On presentation, he was tachycardic (heart rate 105 bpm) but otherwise hemodynamically stable (temperature 36.5°C, blood pressure 106/71 mm Hg, respiratory rate 18, SpO_2_ 100%). Physical examination was unremarkable.

Laboratory evaluation revealed normocytic anemia (hemoglobin 8.1 g/dL), hypoalbuminemia (1.4 g/dL), and mild hyponatremia (sodium 131 mEq/L). White blood cell count was 7.5 × 10^3^/µL with platelets of 305 × 10^3^/µL, creatinine 0.5 mg/dL, lactate dehydrogenase 355 U/L, and liver function tests within normal limits. CD4 count was 196 cells/µL with a viral load of 108,000 copies/mL. HTLV-1 serology was not obtained. An infectious workup was initiated, including blood cultures, stool culture, stool ova and parasites, *Cryptosporidium*/*Giardia* enzyme immunoassay, and *Clostridioides difficile* toxin assay.

Cross-sectional imaging of the abdomen and pelvis demonstrated diffuse abdominopelvic lymphadenopathy, diffuse wall thickening with an ahaustral appearance of the large bowel, asymmetric mass-like thickening in the rectum, and diffuse small bowel wall thickening (Figure 1). Esophagogastroduodenoscopy demonstrated mucosal friability in the stomach and duodenum, while colonoscopy revealed diffuse friability and scattered ulcerations (Figure 2). Biopsies were obtained from multiple sites. The initial infectious workup returned largely unremarkable; the sole positive finding was *Entamoeba coli* on stool ova and parasites; a nonpathogenic commensal that offered no explanation for the severity of his presentation.

**Figure 1.**
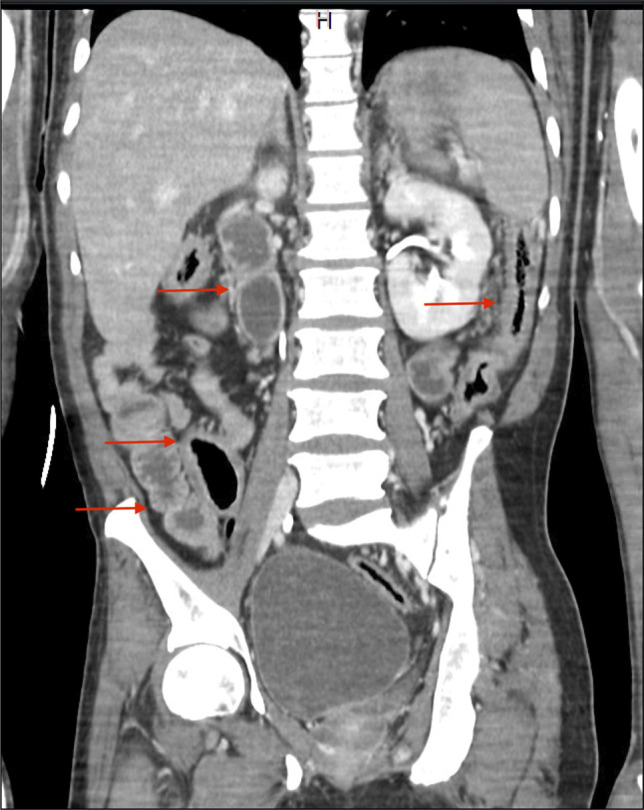
Coronal contrast-enhanced computed tomography of the abdomen and pelvis demonstrating diffuse small-bowel and large-bowel wall thickening with associated abdominopelvic lymphadenopathy.

**Figure 2.**
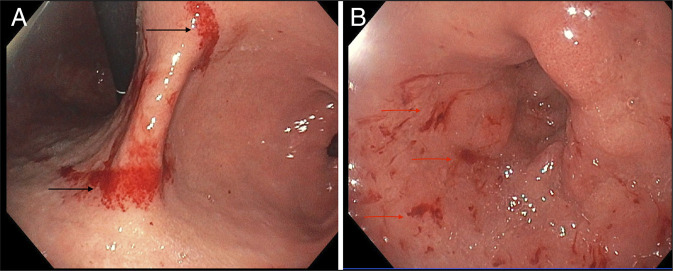
Endoscopic images from esophagogastroduodenoscopy showing mucosal friability in the stomach (A) and duodenum (B).

The following day, the patient's clinical course deteriorated acutely with altered mental status and high-grade fevers, necessitating transfer to the medical intensive care unit for airway protection. Repeat blood cultures grew *Klebsiella pneumoniae* and *Escherichia coli*. Lumbar puncture demonstrated a cerebrospinal fluid white blood cell count of 83 cells/µL with greater than 90% polymorphonuclear predominance and protein exceeding 200 mg/dL, consistent with bacterial meningitis.

At this juncture, comprehensive chart review revealed peripheral eosinophilia (absolute eosinophil count 1.2 × 10^3^/µL) documented 1–2 months before presentation, which had since normalized. The constellation of polymicrobial Gram-negative bacteremia, bacterial meningitis, antecedent eosinophilia, and the patient's epidemiologic background raised strong suspicion for disseminated strongyloidiasis. *Strongyloides* serology was sent. A plan to initiate ivermectin was formulated; however, the patient succumbed to his illness before treatment could be administered. Serology returned positive shortly after death. Intestinal biopsies subsequently demonstrated organisms morphologically consistent with *Strongyloides stercoralis* in the duodenum, stomach, and colon, each associated with acute mucosal inflammation (Figure 3).

**Figure 3.**
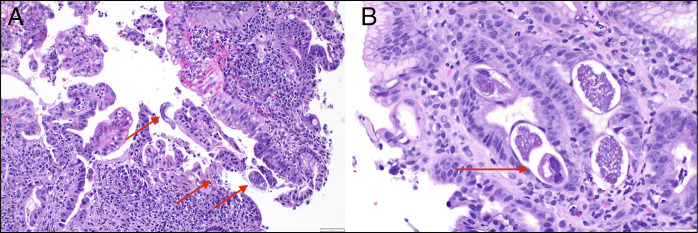
Histopathology showing (A, 60×) duodenal mucosa with neutrophilic and eosinophilic infiltrates and multiple larvae morphologically consistent with *Strongyloides stercoralis* and (B, 40×) gastric mucosa with acute inflammation and cross-sections of an adult female *Strongyloides* worm, both stained with hematoxylin and eosin.

## DISCUSSION

This case illustrates the devastating consequences of unrecognized disseminated *Strongyloides stercoralis* infection in an immunocompromised host and highlights several important clinical lessons for gastroenterologists and internists. Disseminated strongyloidiasis is a life-threatening complication of chronic infection, with very high reported mortality rates when associated with hyperinfection and if left untreated.^3^

Unlike many classic opportunistic infections seen in advanced HIV, severe strongyloidiasis is more strongly associated with corticosteroid exposure and HTLV-1 coinfection than with CD4 depletion alone.^3,4^ Although advanced HIV infection is associated with increased intestinal colonization in endemic regions, disseminated disease has historically been considered paradoxically uncommon.^4^ One proposed explanation is the relative preservation of Th2-mediated immunity central to helminth defense even as CD4 counts decline.^6^ By contrast, HTLV-1 infection disrupts Th2 responses and is strongly associated with hyperinfection and dissemination.^4,7,8^ The principal limitation of this report is the absence of HTLV-1 serology. Because HIV alone rarely drives dissemination, the fulminant course implies an additional permissive factor most characteristically HTLV-1, which reproduces this phenotype by suppressing the Th2 responses that contain *Strongyloides*, although the severe malnutrition documented here (albumin 1.4 g/dL) is a second recognized cofactor.^6–9^ We therefore cannot identify the dominant driver, only that HIV was unlikely to have acted alone. which argues for routine HTLV-1 testing in patients from endemic regions presenting with disseminated disease.

Gastrointestinal strongyloidiasis is protean and often nonspecific.^10^ In the setting of HIV infection, bowel wall thickening and abdominopelvic lymphadenopathy raise concern for lymphoma, Kaposi sarcoma, cytomegalovirus colitis, gastrointestinal mycobacterial infections, inflammatory bowel disease, gastrointestinal malignancy, and acquired immunodeficiency syndrome (AIDS) enteropathy.^11–14^ Endoscopic findings of diffuse mucosal friability and ulceration are similarly nonspecific. Although strongyloidiasis is not classified as an AIDS-defining opportunistic infection, our patient's presentation demonstrates that disseminated disease can occur in HIV-infected individuals with moderate immunosuppression (CD4 196 cells/µL), particularly when compounded by malnutrition.

Critically, antecedent peripheral eosinophilia even if transient should be recognized as a diagnostic clue and should prompt targeted *Strongyloides* testing. In our patient, eosinophilia documented months earlier had normalized by presentation, likely reflecting the transition from chronic infection to disseminated one, during which eosinophil counts paradoxically decrease.^3^

Given the exceptionally narrow therapeutic window associated with hyperinfection, empiric ivermectin should be strongly considered in high-risk patients presenting with unexplained gastrointestinal disease and Gram-negative sepsis while confirmatory testing is pending. Our patient's death before treatment could be initiated underscores that disseminated strongyloidiasis remains a medical emergency in which clinical suspicion must precede diagnostic confirmation.

## DISCLOSURES

Author contributions: GY Amdetsion: Case identification, data collection, literature review, manuscript drafting. R Kwei-Nsoro: data collection, literature review, manuscript drafting and editing. C. Nwabeuze: Revision and drafting of the manuscript. A. Abegunde: Clinical oversight; critical review and editing of drafts; approval of final manuscript. G.Y. Amdetsion is the article guarantor.

Financial disclosure: None to report.

Informed consent for this case report was obtained from the patient's next of kin (father) following the patient's death.

## ABBREVIATIONS:

AIDS: acquired immunodeficiency syndrome
CD4: cluster of differentiation 4
CMV: cytomegalovirus
HIV: human immunodeficiency virus
HTLV-1: human T-lymphotropic virus type 1
SpO_2_: peripheral oxygen saturation
Th2: T helper type 2
